# P-743. Assessing Doxycycline Post-Exposure Prophylaxis (doxy PEP) Engagement in Care at a Midwestern HIV/PrEP Clinic

**DOI:** 10.1093/ofid/ofaf695.954

**Published:** 2026-01-11

**Authors:** Vanessa L Lo, Josh Havens, Sara H Bares, Jennifer M Davis, Kimberly Scarsi, Shawnalyn Sunagawa

**Affiliations:** University of Nebraska Medical Center, Omaha, NE; University of Nebraska Medical Center, Omaha, NE; University of Nebraska Medical Center, Omaha, NE; University of Nebraska Medical Center, Omaha, NE; University of Nebraska Medical Center, Omaha, NE; University of Nebraska Medical Center, Omaha, NE

## Abstract

**Background:**

Doxycycline post-exposure prophylaxis (doxy PEP) has demonstrated sustained effectiveness in multiple real-world settings. However, data on adherence to guideline-directed follow-up for patients receiving doxy PEP remain limited. This study evaluated adherence to guideline-directed sexually transmitted infection (STI) screenings and patterns of patient engagement at the University of Nebraska Medical Center’s Specialty Care Clinic, HIV/PrEP Programs (UNMC SCC).

**Figure:**
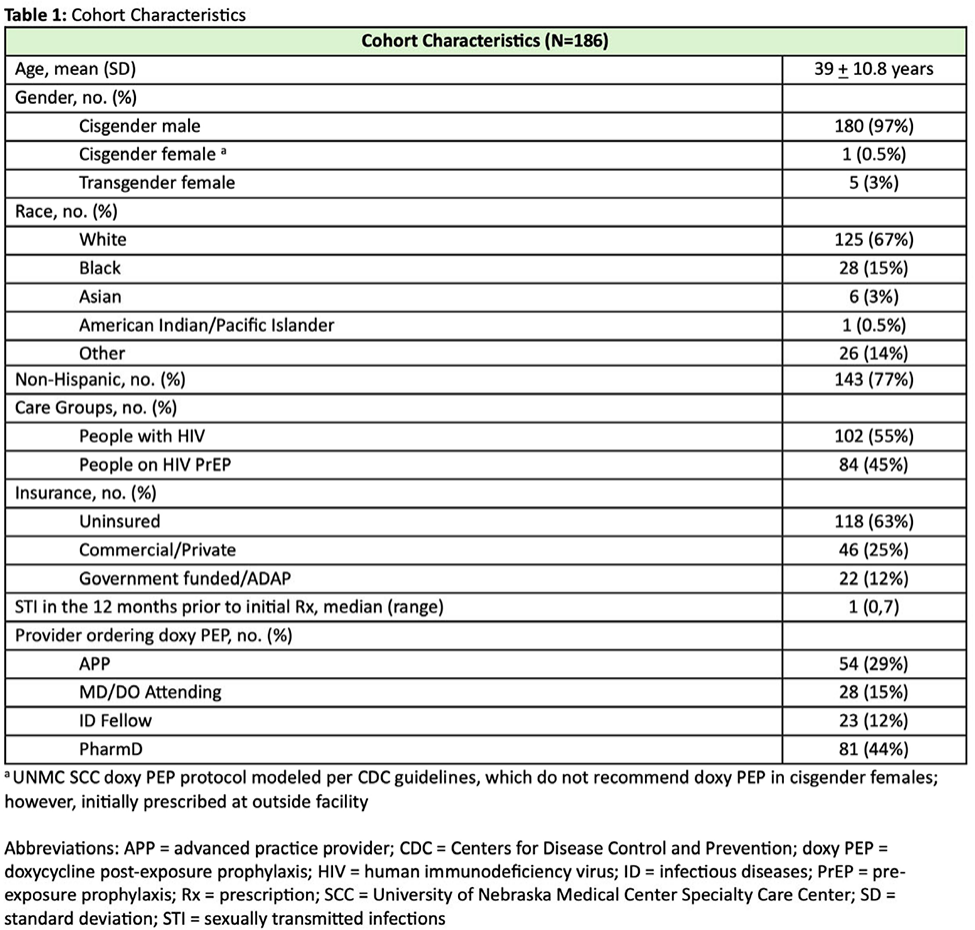

**Figure:**
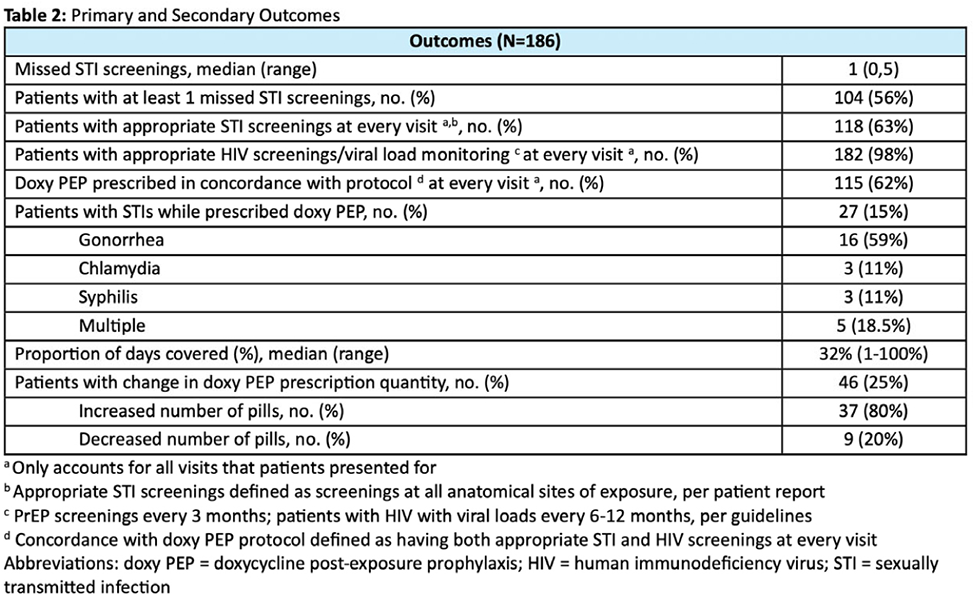

**Methods:**

We conducted a retrospective evaluation of patients who received doxy PEP from UNMC SCC over an 18-month period (June 1, 2023-November 30, 2024). Patients receiving doxycycline for other indications were excluded. The primary outcome was the proportion of patients with missed STI screenings, defined as fewer than 4 screenings in 12 months, per CDC doxy PEP guidelines. Secondary outcomes included number of missed STI screenings, guideline directed STI and HIV screenings, STI diagnoses while prescribed doxy PEP, proportion of days covered (total number of pills prescribed/number of days in study period), and changes in doxy PEP prescription quantity. Descriptive statistics summarized cohort characteristics and outcomes. Chi-square tests assessed associations of select outcomes between people with HIV vs people on HIV PrEP.

**Figure:**
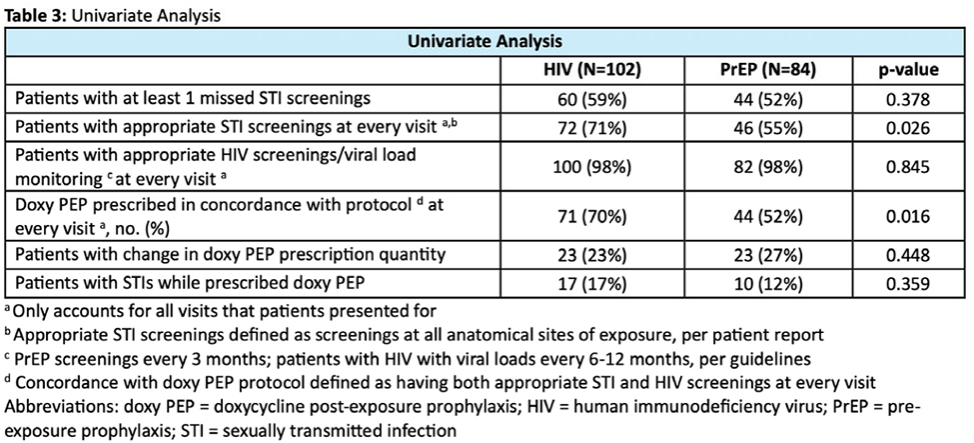

**Results:**

Characteristics of the cohort and prescriptions are listed in Table 1. Primary and secondary outcomes are reported in Tables 2 and 3. Of the 186 patients included in the study, 56% had at least one missed STI screening with a median of 1 (range 0,5) missed STI screening. There was no significant difference in missed STI screenings between care groups (people with HIV vs people on HIV PrEP). A total of 27 (15%) patients were diagnosed with an STI while on doxy PEP, with gonorrhea being the most common (59%). Of those, 17 (16%) were in patients with at least 1 missed STI visit vs 10 (12%) in patients with no missed STI visits (p=0.425).

**Conclusion:**

Engaging patients on doxy PEP in guideline-recommended STI screenings is challenging. Most patients had at least 1 missed STI screening. As utilization of doxy PEP expands, it is necessary to ensure appropriate follow-up and continued engagement in care through routine STI screenings.

**Disclosures:**

Josh Havens, PharmD, Gilead Sciences: Grant/Research Support|Merck: Advisor/Consultant|ViiV Healthcare: Advisor/Consultant Jennifer M. Davis, MD, Viiv: Grant/Research Support Kimberly Scarsi, PharmD, Merck & Co: Grant/Research Support|Viiv Healthcare: Grant/Research Support

